# A Critical Ear: Analysis of Value Judgments in Reviews of Beethoven's Piano Sonata Recordings

**DOI:** 10.3389/fpsyg.2016.00391

**Published:** 2016-03-31

**Authors:** Elena Alessandri, Victoria J. Williamson, Hubert Eiholzer, Aaron Williamon

**Affiliations:** ^1^School of Music, Lucerne University of Applied Sciences and ArtsLucerne, Switzerland; ^2^Centre for Performance Science, Royal College of MusicLondon, UK; ^3^Department of Music, University of SheffieldSheffield, UK; ^4^School of Advanced Study, University of LondonLondon, UK; ^5^Department of Research and Development, Conservatory of Southern SwitzerlandLugano, Switzerland

**Keywords:** music criticism, performance, aesthetic judgment, Beethoven, recordings

## Abstract

What sets a great music performance apart? In this study, we addressed this question through an examination of value judgments in written criticism of recorded performance. One hundred reviews of recordings of Beethoven's piano sonatas, published in the *Gramophone* between 1934 and 2010, were analyzed through a three-step qualitative analysis that identified the valence (positive/negative) expressed by critics' statements and the evaluation criteria that underpinned their judgments. The outcome is a model of the main evaluation criteria used by professional critics: *aesthetic properties*, including intensity, coherence, and complexity, and *achievement-related properties*, including sureness, comprehension, and endeavor. The model also emphasizes how critics consider the suitability and balance of these properties across the musical and cultural context of the performance. The findings relate directly to current discourses on the role of evaluation in music criticism and the generalizability of aesthetic principles. In particular, the perceived achievement of the performer stands out as a factor that drives appreciation of a recording.

## Introduction

The question of what makes a great musical performance has engaged performers, music lovers, philosophers, and scientists for centuries. Since the eighteen century, debate has raged on the subject of taste in the arts (see Hume, 1757), and a century later Helmholtz (1877) published the first empirical work on physiological response to music. The twentieth century saw the development of canons in Western Art Music that placed the evaluation of performance at the core of performers' lives, from exam grades and competition rankings to recording charts and critical reviews by peers and experts. As a consequence, recent research has aimed to clarify the elements that underpin our aesthetic response to a musical performance.

Among music's formal properties that are relevant for aesthetic response are the potential to arouse emotions and the balance between perceived stimulus simplicity and complexity (McDermott, 2012; Juslin and Isaksson, 2014). Complexity, a factor already discussed by Wundt (1874), was given prominence in Berlyne's (1971) inverted U-shaped function that describes the relationship between a stimulus and its hedonic value (Radocy, 1982; North and Hargreaves, 1995, 1996). Aesthetic music properties like complexity come into existence through the interaction between music's objective features and the listener's subjective response, and are thus mediated by the listener's personality, enculturation, familiarity, and expertise as well as circumstantial factors like environment and well-being (Thompson, 2007; McDermott, 2012; Margulis, 2013). In addition, Reber et al. (2004) proposed a framework for the study of aesthetic pleasure that sees processing fluency—the ease in identifying meaning and the physical identity of a stimulus—as a core mechanism underpinning aesthetic experience.

Aesthetic judgments made in educational music contexts have prompted research into the very nature and meaning of performance examinations. Researchers have studied inter-judge reliability (adjudicators' agreement on the quality of a performance) and intra-judge consistency (adjudicators' consistency in evaluating the same performance several times) using holistic and segmented evaluation approaches (Fiske, 1977; Wapnick et al., 1993; Bergee, 1997, 2003; Thompson and Williamon, 2003; Kinney, 2009). While holistic assessments do not offer information on the criteria applied in the evaluation, segmented schemes present an overall judgment conceptualized in terms of specific sub-traits of performance. These sub-traits are developed deductively from literature or criteria commonly used in music schools (Fiske, 1977; Wapnick et al., 1993), or more rarely inductively, from the discussed consensus of music students and pedagogs (Wrigley and Emmerson, 2013; see also Bergee, 2003; Thompson and Williamon, 2003, for examples of mixed deductive/inductive developed schemes). These schemes present a mix of factors that have a criterion-like nature so that an increase in their quality, all the rest being equal, will produce an increase in the quality of the overall performance (e.g., note accuracy, technical security) and of other factors that represent areas of performer competence (e.g., phrasing, dynamics, interpretation). Studies on the reliability and consistency of both holistic evaluations and segmented assessments have offered mixed results, both in terms of degree of agreement and the role of musical expertise. While reliability is typically moderate (Fiske, 1977; Thompson and Williamon, 2003), there is a high level of inter-collinearity between sub-trait ratings in segmented rating scales, a result that has raised doubts regarding the content validity of such schemes (Thompson and Williamon, 2003; Wrigley and Emmerson, 2013).

Taken together, these findings have furthered our understanding of the drivers behind the music evaluation process both within and outside education. However, a comprehensive overview of the qualities that define a great music performance remains elusive (McDermott, 2012). In particular, there is still no consensus on which criteria are used reliably in spontaneous evaluation, how they relate to one another or how they are weighted against one other (Thompson, 2007; Wrigley and Emmerson, 2013). There have been multiple calls for research to explore the nature of value judgments that have emphasized the importance of exploring evaluation by experts in different ecologically valid settings (Bergee, 2003; Gabrielsson, 2003; McDermott, 2012) and the potential benefits of employing systematic qualitative inductive investigations to bring new perspectives on this debate (Wrigley and Emmerson, 2013).

The present study addresses this call directly through an examination of value judgments in written expert criticism of recorded performance. Professional music criticism is a common and relevant form of written response to music. Music critics are seasoned listeners who are musically competent, possess solid semantic and linguistic abilities, and are trained in skills relating to the discussion and evaluation of music outside the academic context. Their writings thus offer important insights into the process of expert performance appreciation and evaluation in the world of commercial recordings: yet this source material has only received marginal attention in empirical music research (Lundy, 2010; Van Venrooij and Schmutz, 2010) and to date no study has investigated the way that critics apply different evaluative criteria.

The first attempt at a systematic and inductive investigation of music performance criticism was carried out by the present authors (Alessandri et al., 2015). A large corpus of recorded performance critical reviews of Beethoven's 32 piano sonatas (*N* = 845) published in the *Gramophone* magazine (1923–2010) was analyzed through a series of data reduction and thematic analyses. This study led to the first empirically developed descriptive model of the content of critics' judgments. The model identified the performance elements that critics discuss in their reviews, distinguishing between musical sound properties, energy level and mechanics of performance (primary descriptors), higher-order impressions of performance in terms of structure, artistic style, dialogue, understanding, character, and emotion (supervenient descriptors), and the value attributed to all these elements (evaluative judgments). The next logical step in this research process was to investigate how primary and supervenient performance descriptors relate to evaluative judgments. Specifically, we examined the ways in which performance descriptors were expressed in terms of positive, negative or neutral valence. Through this secondary examination of the Beethoven reviews we aimed to elucidate how musical appraisals are structured by critics to build value judgments and the reasons they proffer to support their overall aesthetic evaluations.

## Materials and methods

### Source material

During the previous examination of recorded performance criticism (Alessandri et al., 2015) a sample of critical review was collated that encompassed 100 reviews of one or more of Beethoven's 32 piano sonatas, published in the British magazine *Gramophone* between August 1934 and July 2010. This sample was used in the present study as source material.

The 100 reviews comprised 35,753 words, excluding titles, critic names, and recording details. They were written by 10 critics (10 reviews/critic) who had an average period of activity as *Gramophone* reviewers of Beethoven's piano sonatas of over 20 years (see also Alessandri et al., 2014). The review corpus entailed discussion of 56 different pianists and contained at least six reviews for each of Beethoven's 32 sonatas (Table 1).

**Table 1:** **From Alessandri et al. (2015): Corpus selected for the in-depth thematic analysis**.

**Reviewer**	**Reviews (Gramophone issue, page)**
Alec Robertson	Aug'34, p. 29; Oct'35, p. 18; Apr'36, p. 18; Nov'36, p. 17; Feb'37, p. 19; Oct'45, p. 16; Feb'47, p. 8; Feb'48, p. 23; Aug'50, p. 23; Oct'53, p. 22
Roger Fiske	Jul'55, p. 44; Nov'57, p. 17; Oct'58, p. 65; Apr'59, p. 64; Nov'59, p. 67; Nov'59, p. 68; Feb'61, p. 48; Aug'63, p. 31; Jul'84, p. 41; Feb'86, p. 52
Joan Olive Chissell	Mar'69, p. 66; Jun'69, p. 53; Feb'70, p. 54; Dec'70, p. 86; Jun'71, p. 54; Mar'72, p. 74; Mar'75, p. 81; Oct'80, p. 71; Feb'83, p. 52; Jun'92, p. 66
Andrew Porter	Jun'54, p. 42; Oct'54, p. 50; Oct'54, p. 51; Feb'55, p. 56; May'56, p. 49; Nov'56, p. 55; Jun'57, p. 19; Sept'57, p. 17; May'58, p. 16; Feb'59, p. 60
Stephen Plaistow	Dec'61, p. 57; Jun'62, p. 64; Jun'63, p. 36; Mar'64, p. 63; Mar'65, p. 57; Jul'66, p. 47; Aug'79, p. 69; Mar'88, p. 50; Oct'89, p. 98; Jan'02, p. 81
Richard Osborne	Apr'82, p. 66; May'83, p. 49; Dec'83, p. 84; Aug'86, p. 49; Mar'93, p. 73; Sept'95, p. 83; Nov'95, p. 146; Feb'96, p. 75; Nov'00, p. 86; Nov'04, p. 79
Malcolm MacDonald	Aug'54, p. 39; Nov'64, p. 52; Jan'65, p. 59; Mar'65, p. 57; Mar'65, p. 58; Jan'68, p. 84; Jan'70, p. 56; May'81, p. 92; Nov'81, p. 82; Dec'81, p. 84
David J. Fanning	Sept'86, p. 84; Nov'86, p. 78; Sept'88, p. 80; Jun'89, p. 64; Mar'90, p. 69; Sept'90, p. 116; Oct'90, p. 116; Mar'91, p. 85; Apr'92, p. 111; Nov'92, p. 152
Bryce Morrison	May'93, p. 74; Feb'02, p. 63; Dec'02, p. 72; Mar ‘03, p. 63; Jan’05, p. 76; May'05, p. 104; Jun'06, p. 71; Jun'08, p. 81; Jul'10, p. 77(i); Jul'10, p. 77(ii)
Jed Distler	Oct'05, p. 81; Dec'05, p. 97; May'06, p. 90; Sept'06, p. 80; Nov'06, p. 97; Apr'07, p. 92; Jun'07, p. 84; Sept'07, p. 76; Dec'08, p. 103; Oct'09, p. 88

The table reports names of the 10 selected critics and details of the single reviews (issue, page).

Review texts were pre-prepared for the present study by separating performance-related statements from statements that concerned other aspects of the recording product, like the source composition or the recording process (following the findings in Alessandri et al., 2015). Review text that concerned such matters, and therefore did not focus on the nature of the performance, was removed before analysis.

### Analysis

A three-step qualitative analysis protocol was developed based on Guest et al. (2012) Applied Thematic Analysis approach, to examine systematically the valence expressed by critics' judgments and its relationship with their performance descriptions. Applied Thematic Analysis is a methodological framework for qualitative text analysis that shares with grounded theory the requirement for a systematic and transparent analysis protocol (development of codebook, systematic and iterative comparison between themes, and grounding of assertions in the data) but considers the data as paramount when deciding what analytical techniques to apply, without excluding a priori any methodological stance. Here, the three analysis steps focused on (i) the valence expressed by critics' statements, (ii) the relationship between this valence and critics' use of performance descriptors, and (iii) the identification of basic evaluation criteria reliably used in reviews. These steps are described in the following sections.

#### Valence in critical review

Firstly, all the review texts were analyzed independently by two researchers (the first and second authors). The two researchers are both musically competent, but in different instruments (piano and classical guitar, respectively) and at different levels of formal musical education [18 years of training and postgraduate degrees and 14 years of training and Grade 8 (Trinity), respectively], so to provide the viewpoint of two common groups of music review consumers: the professional pianist, who comes with first-hand experience of the repertoire and beliefs concerning how the reviewed works should be performed, and the musically informed reader, who has a solid grasp of the vocabulary but not specific competence concerning the repertoire. Both coders were fluent in English, the language of the reviews, and the second coder was a native speaker, ensuring the comprehension of idioms.

It was decided a priori to use four comprehensive and mutually exclusive valence categories during coding: ***positive***, ***negative***, ***neutral***, and ***unclear***. After a preliminary pilot analysis phase a fifth category, ***mixed***, was added to capture text units that entailed both positive and negative valence. Definitions for the five valence codes and examples of coded text are shown in Table 2.

**Table 2:** **Codebook used for the analysis of valence content in critical review**.

**CODE**	**Definition**	**Example**
Positive	Statements with clear positive valence	“It is played magnificently. Schnabel gives a most dramatic reading of the work, leaving us in no doubt as to its essential bigness” (Robertson, August 1934, p. 29)
Negative	Statements with clear negative valence	“The section of the slow movement has a certain beauty which I feel Schnabel spoils by too dynamic a treatment” (Robertson, April 1936, p. 18)
Mixed	Statements entailing both positively and negatively loaded parts, which cannot be taken apart without losing the meaning of the text unit	“The last movement, needless to say, is played in the grand manner and is undeniably exciting, but without the fine nuances of phrasing and articulation Gieseking gives us” (Robertson, October 1953, p. 22)
Unclear	Statements: (i) for which it is not clear if they entail some valence or not; or (ii) which seem to entail some valence, but for which it is not possible to decide if this is positive or negative	“The final fugue is something more than a struggle against appalling odds” (Fiske, August 1963, p. 31)
Neutral	Statements that are purely descriptive, they entail no valence	“Kempff, by the way, does not follow the Schnabel edition, so that there are some textual differences in the two performances” (Robertson, November 1936, p. 17)

Each researcher coded the whole corpus according to the five pre-defined categories. Segmentation was performed at the smallest multiple-clause level necessary to perceive a clear valence in the text. Upon completion of the coding, agreement level was computed. Statements that presented a lack of agreement were discussed between the two coders; researchers took turns in explaining their reasoning behind the coding of each statement. Details on agreement are reported in the relevant results section. This process led to a revised version of the coded documents agreed upon by both researchers that offered a valence-based categorization of critical statements.

#### Relationship between valence and performance descriptors

For the next step, text that was coded as ***unclear*** or ***neutral*** was disregarded. Separate quote lists of valence loaded statements (i.e., ***positive***, ***negative***, or ***mixed***) were retrieved for each one of the primary and supervenient descriptors detailed by Alessandri et al. (2015). Main descriptors included Musical Parameters, Energy, and Technique (primary); Style, Structure, Character, Understanding, Emotion, Dialog, and Performer Qualities (supervenient). Table 3 provides a full list of descriptors together with co-occurrence frequencies between descriptors and valence loaded statements. This overview offered us first insights on the structure critics used in building value judgments in terms of simple valence proportion. Based on this, separate quote lists for each primary and supervenient descriptor and sub-descriptor (30 co-occurrence lists, retrieved with ATLAS.ti software, version 6.1^1^) were analyzed by the first author to identify the qualities of performance descriptors that were adduced by critics to support their judgments. For each descriptor (e.g., Dynamics, Emotion), the aim of the analysis was to clarify the relationship between the valence expressed in the statement and the property identified by the descriptor. This was done by asking, for each statement, what critics praised (positive statements) or criticized (negative statements) about the descriptor. For mixed valence statements, the positive and negative parts had to be sorted out and analyzed on a single case basis. This led to the identification of a set of descriptor qualities recurrently discussed in reviews as adding to the value of a performance, known henceforth as “value adding qualities.”

**Table 3:** **Co-occurrence table between main and sub-descriptors used in the analysis of critics' value judgments and positive, negative, and mixed valence loaded statements**.

**Descriptor Family**	**Descriptor**	**Sub-descriptor**	**Co-occurrence with valence loaded statements**			
			**Positive**	**Negative**	**Mixed**	**TOTAL**
Primary	**Musical Parameters**		108	94	52	**254**
		***Tempo***	40	30	17	**87**
		***Color***	29	9	14	**52**
		***Dynamics***	10	21	12	**43**
		***Rhythm***	10	19	9	**38**
		***Articulation***	14	16	6	**36**
		***Expressive timing***	9	13	10	**32**
	**Energy**		84	33	27	**144**
		***Tension***	8	5	1	**14**
	**Technique**		68	23	13	**104**
		***Virtuosity***	15	1	0	**16**
Supervenient	**Style**		175	96	65	**336**
		***Expressive***	9	7	5	**21**
		***Historical***	9	6	5	**20**
	**Structure**		121	52	27	**203**
		***Balance***	37	11	5	**53**
		***Journey***	19	9	4	**32**
		***Emphasis***	10	7	6	**23**
	**Character**		100	45	30	**175**
	**Understanding**		88	24	31	**143**
	**Emotion**		48	21	17	**86**
	**Dialog**		31	9	8	**48**
	*Performer Qualities*	***Performer Style***	102	45	34	**181**
		***Control***	28	5	7	**40**
		***Care***	16	12	6	**34**
		***Sensibility***	19	3	1	**23**
		***Spontaneity***	5	3	2	**10**
		***Intention***	5	0	0	**5**
		***Performer Understanding***	54	18	17	**89**
		***Performer Emotion***	17	5	11	**33**
		***Performer Character***	15	5	5	**25**

Main descriptors are given in bold, sub-descriptors in bold-italic. Full discussion of each descriptor is reported in Alessandri et al. (2015).

#### Performance evaluation criteria in critical review

Finally, the emergent value adding qualities were analyzed by the first author to identify underpinning higher-order evaluation criteria. Here, a procedure proposed by Beardsley (1968) was adopted for the establishment of the basic criteria: for each value adding quality the reader asks “Why is this positive?” and “How does it add to the value of the performance?” This process was repeated until a property was reached, whose positive value could no longer be explained by appealing to features of the work itself (understood broadly to embrace features the work represents, suggests, or symbolizes). For example, “a rich and resonant sound” can be said to be positive in that it renders the performance more intense. Explaining why resonant sound is good can be done by appealing to a more general principle: intensity is desirable in the performance. Explaining on the other hand why intensity is desirable would need an explanation that goes beyond what is entailed in the performance. Therefore, following Beardsley's process, intensity can be taken as a higher-order evaluation criterion. The application of this procedure to all the value adding qualities found in step-two of the analysis led to the development of a model of the performance evaluation criteria used in our critical review corpus.

## Results

### Valence in critical review

#### Inter-coder agreement

In total, 943 text segments were coded across the 100 reviews. Percentage of agreement between the two coders was 76.03%, Cohen's Kappa = 0.65 (*p* < 0.001), 95% CI (0.62–0.69), which represents a substantial level of agreement between coders (Landis and Koch, 1977). The discussion of discrepancies between coders revealed that disagreements were mainly due to (i) ambiguities in the reading of the text, for instance concerning comparative judgments or conditional statements (statements in the form “if you like X you will like this performance”); (ii) nuances in the interpretation of the value component of words, partly due to the different perspectives and levels of familiarity with the repertoire the coders had (e.g., the characterization of a performance of Op. 2/1 as “Haydnish” has a different valence once the reader knows that this sonata was dedicated to Haydn by Beethoven); and (iii) misjudgment of word meaning or idiomatic expressions linked to one coder being a fluent but not native English speaker (e.g., terms like “fastidious” have a clear and consistent negative connotation in Latin languages but not so in English). Many such discrepancies were solved by creating three *ad hoc* rules:

(i) Comments partly unclear and partly clearly positive/negative were assigned the valence of the clear fragment. Example:
  “…there is plenty of matter for discussion in Schnabel's interpretations, besides lots for any pianist to learn and profit from” (Robertson, April 1936, p. 18^2^).
“Matter for discussion” could be interpreted as either positive or negative. The second part of the sentence is however clearly positive. The whole segment was then coded as positive.
(ii) Comments in the form “If you like property X, then you will like/dislike performance P” or its variations were coded as ***mixed***, in that they suggest that the value of the performance is dependent upon the listener perspective or taste. Example:
  “If you like Beethoven's dynamics undefined and a presentation of him in a thoroughly unbuttoned mood you will warm to Medtner's interpretation of the first and last movements of the Apassionata” (Robertson, February 1947, p. 8).
(iii) Statements that compared two performances were coded according to the valence of the terms used. Example:
  “I preferred Ashkenazy's for its stronger voltage and drive” (Chissell, February 1970, p. 54).
This statement could be seen as positive (for Ashkenazy) or negative (for the performer set against Ashkenazy). The terms used (“preferred,” “voltage,” and “drive”) carry a positive valence, therefore the sentence was coded as overall positive.

The application of these three rules to the 23.97% of un-agreed statements (*n* = 226) led to complete agreement between the two coders with the exception of two statements: these two statements were thus excluded from subsequent analyses. Therefore, the following results are based on a sample of 941 text fragments.

#### Valence expressed by review statements

The majority of critical review text (87.57%) emerged as clearly valence loaded (***positive***, ***negative*** or ***mixed***), with a bias toward ***positive*** comments. ***Neutral*** statements were rare and a small amount of comments were coded as ***unclear*** (Table 4).

**Table 4:** **Frequency of code occurrence for the five valence categories**.

**Valence category**	**Number of coded text segments**	**Percentage (%)**
Positive	468	49.73
Negative	221	23.49
Mixed	135	14.35
Neutral	69	7.33
Unclear	48	5.10
Total	941	100

**Figure 1:**
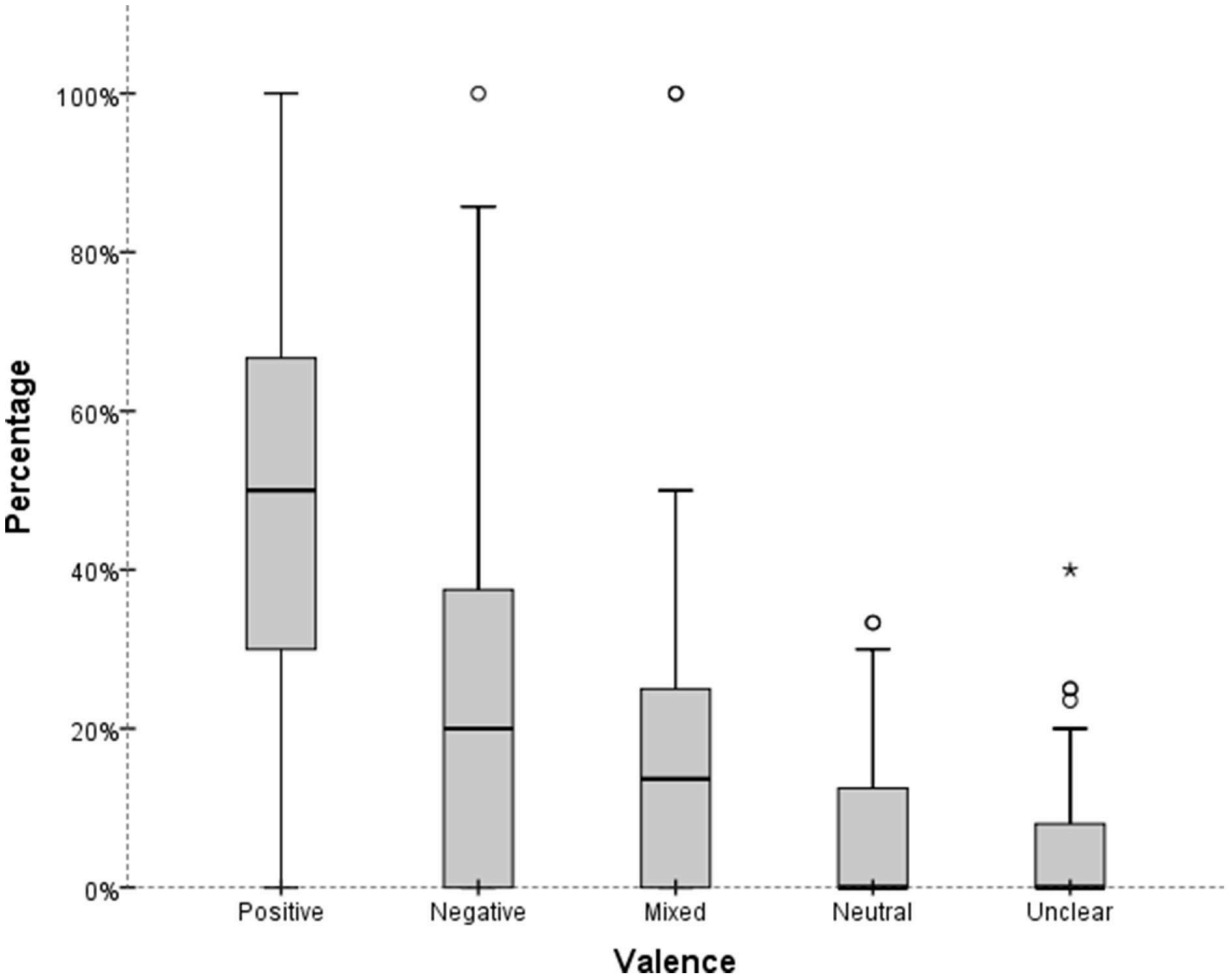
**Distribution of valence statements within reviews**. Small circles and star show single case outliers, that is, observations located more than 1.5 (circle) and 3 (star) times the IQR away from the upper quartile.

Valence in critical review was found to be expressed both explicitly through purely evaluative terms (e.g., good or excellent vs. poor or unduly) and implicitly through the use of valence loaded descriptors (e.g., “nimble fingers” vs. “overtaxed fingers”). An analysis of valence distribution within single reviews showed that reviews were characterized by a juxtaposition of ***positive*** and ***negative*** judgments: On average (median), each review entailed 50.00% (IQR = 31.50–68.50) of ***positive***, 20.00% (IQR = 1.00–39.00) of ***negative***, and 13.66% (IQR = 1.16–26.16%) of ***mixed*** statements (Figure 1). Across the 100 reviews, seven reviews entailed only positive statements, and just one review encompassed only negative ones. The latter is a very short review of Backhaus's Pathétique and Moonlight sonata, entailing just one performance-related sentence:

“The performances I found disappointing, and I would suggest there exist a number of couplings of these two sonatas that are superior to this one” (Plaistow, June 1962, p. 64).

### Relationship between valence and performance descriptors

Following the analyses of the co-occurrences between valence loaded statements (***positive***, ***negative***, or ***mixed***) and primary and supervenient descriptors, 39 qualities of performance descriptors were identified, recurrently adduced in critical review as reasons to support evaluative judgments. In what follows, the names of main and sub-descriptors are presented in bold (first level of hierarchy) and bold italic (second and third levels of hierarchy) and the related value adding qualities are presented in italics. For each performance descriptor, qualities are reported that were mentioned at least three times in the text, together with examples from the reviews. Numbers in parentheses after value adding quality names show the frequency with which each quality was coded in the text for the given descriptor.

#### Valence of primary descriptors

Primary descriptors entail comments on parameters of the musical sound (***Tempo***, ***Color***, ***Dynamics***, ***Rhythm***, ***Articulation***, and ***Expressive Timing***), level of **Energy**, and mechanics of delivery (**Technique**). A comparison with the findings of Alessandri et al. (2015) shows that 65.37% of these comments (470 out of 719) were connected to a valence loaded statement. The percentage was the highest for **Energy** (79.00%) and lowest for **Technique** (29.14%). Six recurrent value adding qualities were common to several primary descriptors: *Appropriateness, Clarity, Variety, Energy, Control*, and *Accuracy*. In addition, a series of descriptor-specific qualities was found. **Energy** and its sub-descriptor ***Tension*** emerged as basic value adding qualities in their own right. **Musical Parameters** and in particular ***Tempo*** were discussed as relevant to different aspects of performance like *Fluency, Energy, Clarity*, and *Affective power*. In line with a previous analysis by the authors (Alessandri, 2014) ***Expressive Timing*** was more often criticized than praised by critics. Finally, the discussion of **Technique** focused on the two aspects of (i) technique as an aid to interpretation and its compliance with the music's demands, and (ii) the value of technique as a basic positive quality of performance in terms of *Brilliance* and *Virtuosity*.

#### Musical parameters

***Tempo:*** Comments on speed (beat frequency) on global level.

*Fast Tempo* (28): A fast tempo was at times praised or desired. Reasons given referred to the influence of ***Tempo*** on the performance *Fluency, Balance*, and *Energy.*
  “…his [faster] tempo helps to keep the line buoyant here and the material of the episodes belonging to the rest” (Plaistow, August 1979, p. 69).
*Appropriateness* (22): ***Tempo*** in line with the music character or demands.
  “Gieseking tears off the first movement of the Pathétique to a tremendous pace, perhaps a little too fast to convey its tragic grandeur” (Fiske, November 1957, p. 17).
*Slow Tempo* (17): A slow ***Tempo*** was at times praised or (more often) desired. Reasons given referred to the influence of ***Tempo*** on the performance *Clarity, Affective power*, and *Control.*
  “The Scherzo proceeds a shade too fast for the woodwind-like staccato to register fully” (Distler, October 2005, p. 81).
*Balance* (13): Relationships between ***Tempi*** that add to the coherence of the overall interpretation.
  “The first movement—until the rest has been heard—may perhaps be thought a shade slow, not ebullient or sparkly enough; but a bewitching performance of the Scherzo, at a very lithe gait, gives retrospective point to the earlier speed” (Porter, November 1956, p. 55).

***Color:*** Comments on timbral and textural sound properties.

*Richness* (19): Resonant, warm, deep sound, in particular at *f* and *ff* dynamic levels.
  “His launching of the work gives warning of its stature—the fortissimo opening chords are richer in tone than Brendel's” (Chissell, March 1972, p. 74).
*Variety* (16): Use of timbral range.
  “Wührer's limitation …lies in a certain sameness of tone-color” (Porter, June 1957, p. 19).
*Control* (5): Ability to use and control timbral qualities.
  “There is enjoyment to be had from hearing the textures so adroitly controlled” (Fanning, September 1988, p. 80).
*Appropriateness* (5): Timber in line with the music character.
  “A beautiful cantabile distinguishes his playing of the F sharp minor melody beginning at bar 27, infinitely seductive but out of place here” (Robertson, November 1936, p. 17).

***Dynamics:*** Comments on the loudness of the musical sound.

*Variety* (22): Wide range of dynamic levels. Emphasis on the use of pp for expressive ends.
  “…because of a reluctance to drop to piano or pianissimo in the last, the Waldstein has difficulty here in catching fire” (MacDonald, January 1965, p. 59).
*Accuracy* (14): Precision in differentiation between dynamic levels and following score indications.
  “I do not understand …why so many of Beethoven's dynamics in the variation movement—indications of pianissimo especially—are ignored” (Plaistow, January 2002, p. 81).

***Rhythm:*** Comments on rhythm understood as patterns of accents (Cooper and Meyer, 1960).

*Steadiness* (14): Unwavering and even pulse.
  “Her way with the giant fugal finale, too …includes an end sadly out of rhythmic kilter” (Morrison, July 2010, p. 77).
*Energy* (9): Strong and vital rhythmic impulse.
  “…the first movement …is full of superb rhythmic energy” (Robertson, April 1936, p. 18).
*Clarity* (7): Precision in the delivery of rhythmic patterns.
  “…he takes a few bars to define the stinging dotted rhythm with Brendel's clarity” (Chissell, March 1972, p. 74).
*Control* (3): Ability to use and control pulse and rhythmic patterns.
  “My other serious quibble concerns Foldes's control of the rhythmic flow of the music” (Plaistow, December 1961, p. 57).

***Articulation:*** Comments on the way in which successive notes are connected.

*Emphasis* (14): Effective use of accents and *sforzandos* that adds to the performance *Tension*, generates drama and urgency or evidences structural relationships.
  “…the development section's relentless left-hand arpeggios gain urgency through unusual accentuations” (Distler, September 2007, p. 76).
*Clarity* (9): Precise and well-differentiated articulations.
  “The difficult fourth movement …is played with great power and absolute clarity of articulation” (Robertson, February 1948, p. 23).
*Variety* (4): Wide range of articulations.
  “For breathtaking variety of articulation, listen to Op. 2 No. 1's Largo appassionato” (Distler, December 2005, p. 97).
*Accuracy* (3): Exactness in following score indications.
  “…but there, too, lies the snag. It's only necessary to play the first few bars of the Pathetique to discover a ‘this is my Beethoven’ attitude. Dotted notes …get their dots as it were moved from their sides to their tops” (Chissell, June 1971), p. 54.
*Lightness* (3): Light touch.
  “Schnabel …gives the ubiquitous semiquavers a light and almost fantastic touch” (Plaistow, March, 1964, p. 63).

***Expressive Timing:*** Comments on speed at the local level; variations from an underlying beat frequency.

*Fluency* (8): The use of ***Expressive Timing*** was criticized for marring the unity and flow of the performance.
  “…he makes a ritardando at the end of the A major's first movement …of dimensions far too large for the movement as a whole to sustain without structural unbalance” (Plaistow, December 1961, p. 57).
*Affective power* (7): The use of ***Expressive Timing*** was praised (or desired) for adding to the performance emotional intensity.
  “Many will find the first movement of the Moonlight also rather lacking in feeling—there is almost no rubato” (Fiske, July 1984, p. 41).
*Accuracy* (6): Exactness in following score indications.
  “The only inconsistency here is the surprisingly sudden plunge in the triumphant final return of the fugue subject (in left hand octaves) instead of the composer's prescribed poco a poco animation” (Chissell, June 1969, p. 53).
*Appropriateness* (5): Use of ***Expressive Timing*** in line with music style and character.
  “K., lovely though his playing is, makes some parts of the music which are decorated sound like a Chopin nocturne rubato and all!” (Robertson, November 1936, p. 17).

**Energy:** Characterizations of the performance that suggest strength and vitality.

Both **Energy** and its sub-descriptor ***Tension*** presented a homogeneous construct in critical review: they were both praised as basic value adding qualities of performance.
  “…his playing is never less than acute, his energy coursing like electricity from point to point, from pylon to pylon” (Morrison, June 2006, p. 71).
*Appropriateness* (11): When a performance was criticized for being too energetic or tense this was explained in terms of appropriateness to the music character.
  “Among the late sonatas, tension and severity serve Gulda's Opp. 109 and 110 less well than in his remarkably concentrated Hammerklavier” (Distler, September 2006, p. 80).

**Technique:** Comments on the mechanics of performance delivery (e.g., fingering, use of pedal or realization of ornaments).

*Appropriateness* (23): Technique put at the service of the music, as an aid in the communication of the musical message and realization of other performance properties (e.g., *Energy, Variety, Affective power*).
  “Sheppard is never less than eloquent, his outsize technique and personality always at the composer's service” (Morrison, June 2006, p. 71).
*Clarity* (21): Technical precision.
  “…Freire, like Claudio Arrau, takes trouble to make the first movement's rapid left-hand figurations clear and distinct” (Distler, September 2007, p. 76).
*Assuredness* (11): Conveyance of mastery and command of technical challenges.
  “Watt's technique is very rarely embarrassed by Beethoven's demands” (Fanning, September 1988, p. 80).
*Energy* (9): An energetic and exciting technical delivery.
  “…some might prefer Richard Goode's more impetuous, angular fingerwork (Distler, June 2007).
*Brilliance* (7): Basic value adding quality of **Technique**.
  “…Gilels plays the music with …great technical brilliance” (Osborne, August 1986, p. 49).
The sub-descriptor of **Technique**, ***Virtuosity***, was used as a basic value adding quality of the performance.
  “He plays the jolly little scherzo and the difficult finale with much virtuosity—the right and only way” (Robertson, April 1936, p. 18).

#### Valence of supervenient descriptors

Supervenient descriptors included comments on artistic **Style**, **Structure**, **Character**, **Understanding**, **Emotion**, and **Dialog**. Supervenient descriptors build on primary descriptors in that they relate to the way primary descriptors are used. Out of the 1404 occurrences of supervenient descriptors identified in the corpus (Alessandri et al., 2015), 1016 (72.36%) were used in valence loaded statements.

All the value adding qualities found in primary descriptors were relevant for supervenient descriptors. Supervenient descriptors were, however, more varied and metaphorical in nature; the valence of these statements was strongly shaped by the use of valence laden terms in the characterization of performance. For example, in artistic **Style** performances were characterized as “deft,” “muscular,” or “dainty” vs. “immature,” “brutish,” or “emasculated.”

The value adding quality *Appropriateness* emerged as particularly relevant for the evaluation of **Character**, **Style**, and **Emotion**. A sub-group of supervenient descriptors entailed comments on **Style**, **Character**, **Emotion**, and **Understanding** that focused on qualities of the performer rather than on the performance itself (*Performer Qualities* in Alessandri et al., 2015). These comments were strongly bound with evaluative judgments. Five sub-themes in this group emerged as value adding qualities in their own right, almost always praised by the critics (***Performer Understanding***, ***Control***, ***Care***, ***Sensibility***, and ***Spontaneity***). This group of sub-descriptors is discussed separately at the end of this section.

**Style:** Comments describing manners of execution.

*Appropriateness* (31): Suitability of the style given to the music piece.
  “On the plus side, his muscular, energetic pianism suits middle-period Beethoven's bravura qualities” (Distler, October 2005, p. 81).
*Control* (13): Command over the music demands.
  “…his performances have won praise for their distinctive restraint” (Morrison, May 1993, p. 74).
*Simplicity* (12): Straightforward and unaffected performance.
  “…if you like this music without tricks and mannerisms, you will like this” (Fiske, April 1959, p. 64).
*Finesse* (11): Delicacy and high skill in delivering the performance.
  “Try Op. 28's finale for an ultimate pianistic and musical finesse” (Morrison, June 2008, p. 81).
*Variety* (10): Way of playing that emphasizes richness of musical elements (e.g., patterns, harmonies, timbers, characters).
  “And who'd have expected such an affectionately nuanced Op. 54, Op. 28 or Pathetique slow movement? (Distler, September 2006, p. 80).
*Effort* (8): Hard work and determination that are applied to the performance.
  “…the issues are brought out into the open, identified, and worked out with great rigor” (Osborne, August 1986, p. 49).
*Breadth* (5): Relaxed, poised, unhurried way of playing.
  “Surely it needs a bit more time to smile, even to breathe” (Chissell, March 1975, p. 81).
*Lightness* (4): “Light” vs. “heavy” performance is praised.
  “The first movement of the “Pathetique” is heavy” (Porter, February 1955, p. 46).
*Steadiness* (4): Firm and balanced performance.
  “Paik also orchestrates the Tempest Sonata's middle movement textures to haunting, rock-steady effect” (Distler, October 2005, p. 81).

***Style Expressive:*** Comments that characterize the manner of execution through the term “expressive” and derivatives (in line with the distinction between uses of the term “express” as explored in Alessandri, 2014).

*Affective power* (12): Performance praised as *expressive* to indicate an intense and effective communication of (unspecified) inner states.
  “…the playing is very expressive” (Fiske, August 1963, p. 31).
  *Variety* (9): Use of expressive inflections in terms of dynamics, color, and structural *Variety* as expressive tools.
  “The slow movement left me wishing that he had not relied so much for expression on rhythmic flexibility, but had sought it instead in a melodic contour shaped by subtle dynamic gradation, pure and simple” (Chissell, June 1971, p. 54).

***Style Historical:*** Manner of execution linked to different practices and historical periods.

*Appropriateness* (14): Style is in line with the music's compositional background and performance practices. These comments require musical knowledge on the part of the reader for the implied valence to become clear.
  “K., lovely though his playing is, makes some parts of the music …sound like a Chopin nocturne rubato and all!” (Robertson, November 1936, p. 17).
*Romantic* (4): Romantic approach is praised, as it adds to *Energy* and *Affective power*.
  “I like to think that his way of allowing such pages to erupt in a blaze of romantic fire …would have won Beethoven's hearty applause and approval” (Morrison, March 2003, p. 63).

**Structure:** Comments on how the performer portrays the design of the music.

*Variety* (30): Portrayal of the music structure highlights contrasts and celebrates the richness of musical details.
  “…the variety of perspectives, from huge vistas to tiny units …all this is Beethoven's doing, but it does not exist without his perceptive interpreter” (Plaistow, August 1979, p. 69).
*Direction* (17): Portrayal of the musical events conveys directionality and shows how elements build together.
  “Schnabel's great gift …of letting us perceive the growth and design of the music stands him in good stead” (Robertson, April 1936, p. 18).
*Clarity* (11): Transparent presentation of musical structure.
  “…the playing of the part-writing in the prestissimo is beautifully clear” (Robertson, February 1937, p. 19).
*Breadth* (8): Spacious and relaxed portrayal of structure.
  “…there were times in the variations when I felt the need for …a more relaxed presentation of events” (Plaistow, June 1963, p. 36).
*Control* (6): Command over the different musical patterns and their presentation.
  “…Gilels' mastery of the music's asymmetric lines” (Osborne, December 1983, p. 84).

***Balance:*** Music portrayal stresses coherence and unity. This sub-theme emerged as value adding quality of performance.

“…there are moments in Op. 106…where he achieves a greater sense of rapport” (Morrison, July 2010, p. 77).

***Journey:*** Characterization of the music portrayal as a dynamic process.

*Fluency* (14): Music portrayal perceived as smooth, fluid, freely flowing.
  “…the first movement needs a stronger and more continuous flow” (Chissell, June 1969, p. 53).

***Emphasis:*** Music portrayal gives prominence to selected structural elements in an effective way. This sub-theme also emerged as value adding quality of performance.

“…Gelber is again finding sunshine in every diatonic seventh, storm-clouds in every minor triad, and the broader lines of thought which distinguish Beethoven from your average Early-romantic are little in evidence” (Fanning, June 1989, p. 64).

**Character:** Characterizations of the performance in terms of mental and moral qualities of an individual or of an atmosphere.

*Appropriateness* (75): Suitability of the character to the music piece.
  “…perhaps the Pathetique's Adagio is not given quite all its inherent dignity” (MacDonald, May 1981, p. 92).
*Energy* (44): Character conveys strength and intensity. Focus on drama and urgency.
  “If anything is missing, it is the sense of tragic pathos” (Osborne, November 2000, p. 86).
*Mystery* (25): Character suggests states and atmospheres linked to transcendental experiences.
  “It is afterwards, in the variations, when the light should dissolve into one that is not of this world, that chinks of common daylight reappear to disturb us” (Porter, October 1954, p. 51).
*Elegance* (16): Gracious, charming character.
  “Backhaus suddenly achieves that sparkling elegance which is a mark of his playing at its best” (Porter, June 1954, p. 42).
*Character* (5): Performance praised for having character (no character specified).
  “…try Op. 79, which can rarely have been given more ebulliently of characterfully” (Morrison, June 2006, p. 71).
*Poise* (4): Character suggests calm and restraint.
  “It gets off to an unsatisfactory start: the exposition …inclines to be hectic” (Plaistow, June 1963, p. 36).
*Risk* (4): Character suggests exposure to danger.
  “…his imagination, I feel, sparks at low voltage …The Allegro molto holds no hint of recklessness” (Porter, September 1957, p. 17).

**Understanding:** Comments on performance qualities that reflect reasoning and use of intellect.

*Insightfulness* (28): Intellectually stimulating performances. Focus on insights, meaningfulness, ambiguity, and fantasy.
  “Goode and Ashkenazy each offer illuminating insights” (Fanning, March 1990, p. 69).
*Thoughtfulness* (12): Rational quality of the performance, reflecting mental focus on key attributes.
  “Yet do not think that this is less than a thoughtful and remarkable performance” (Porter, October 1954, p. 51).
*Clarity* (12): Cogency and lucidity of the performance.
  “At times it is a model of lucidity, arguments and textures appearing as the mechanism of a fine Swiss watch must do to a craftsman's glass” (Osborne, December 1983, p. 84).

**Emotion:** Comments on affective states.

*Appropriateness* (21): Suitability of the expressed emotion.
  “…he fails also to discover the full peace and ethereal quality of the final movements” (Robertson, August 1950, p. 23).
*Affective power* (19): Emotional intensity (no emotion specified).
  “And what a debate it is, substantial and charged with feeling” (Osborne, February 1996, p. 75).
*Poise* (10): Feeling of calm and inner balance.
  “…there are whole sonatas …which in their poise, and lack of haste, are utterly convincing” (MacDonald, January 1970, p. 56).

**Dialog:** Comments on the communicativeness of the performance.

*Clarity* (27): Direct and effective.
  “…the fugal argument is also expounded with a quiet, posed clarity that many an artist needs a life-time to achieve” (Chissell, June 1969, p. 63).
*Sophistication* (23): Refined and beautiful.
  “…he cannot command the poetry of Solomon's wonderful interpretation” (Fiske, July 1955, p. 44).

#### Performer qualities

On average, 81.86% of *Performer Qualities* statements identified in Alessandri et al. (2015) were valence loaded. This percentage reaches above 95.00% for the sub-descriptors of ***Performer Style***: ***Control*** and ***Sensibility***. ***Performer Understanding*** and four out of five sub-descriptors of ***Performer Style*** emerged as value adding qualities in their own right.

***Performer Style****:* Comments on performers' attitude toward or approach to the work. All its sub-descriptors except ***Intention*** were praised as value adding qualities of performance.

*Control* (40): Conveyance of a feeling of aesthetic and technical command over the performance.
  “Beethoven interpretation as vivid and apparently effortless as Brendel's is to be prized” (Plaistow, August 1979, p. 69).
*Carefulness* (34): Attention and rigor in dealing with musical elements.
  “These are both fine, scrupulously judged performances. Every detail has been well-considered” (Porter, May 1958, p. 16).
*Sensibility* (23): Sensitivity to the presence and importance of musical features.
  “In Op. 110 he is most exquisitely sensitive to the phrases” (Porter, October 1954, p. 51).
*Appropriateness* (10): Suitability of the style given the musical demands.
  “For a composer with a backbone like Beethoven's, Barenboim strikes me as always a little too ready to yield” (Chissell, June 1969, p. 53).
*Dedication* (10): Commitment and respect toward the musical piece.
  “Solomon played this movement with immense reverence, as though he thought it the greatest piano music in existence; his performance is an occasion” (Fiske, November 1959, p. 68).
*Spontaneity* (10): Open, natural, instinctive approach to the music.
  “The Adagio from Op. 27 No. 2 is supremely natural and unstudied” (Morrison, December 2002, p. 72).
*Assuredness* (8): Determination and certainty in the delivery of the performance.
  “…in the finale the fugue goes particularly well, Miss Donska achieving the flow and conviction not altogether conveyed earlier on” (MacDonald, November 1964, p. 52).
*Effort* (7): Rigor, work, and determination in the preparation and delivery of the performance.
  “Hear his concentration toward the end of the slow movement” (Fanning, September 1990, p. 116).

***Performer Understanding:*** Comments on performers' comprehension of the music and discernment and imaginative power in its realization. The ***Performer Understanding*** was praised as value adding quality of performance. When criticized, it was discussed in terms of personal preferences or misunderstanding between performer and listener.

“…it is Ashkenazy …whose imaginative penetration makes you the more aware of the composer's breath-taking revelations” (Chissell, February 1970, p. 54).

***Performer Emotion:*** Comments on the performer's affective states.

*Affective power* (19): Performer's emotional involvement with the music.
  “Serkin plays with very deep feeling” (Chissell, March 1972, p. 74).
*Appropriateness* (8): Performer's emotions are suitable to the music in terms of their type and intensity.
  “…he plays it with the right tender feeling” (Robertson, February 1937, p. 19).
*Poise* (6): Emotional control and calm during the performance.
  “In Op. 110 Serkin seems to have regained poise” (Plaistow, October 1989, p. 98).

***Performer Character:*** Comments on mental and moral qualities of the performer.

*Appropriateness* (14): Performer's character suitable to the music piece.
  “…did he really intend to be so severe with the Moonlight's central allegretto?—not so much a “flower between two abysses” (Liszt), more a clump of nettles” (Fanning, November 1986, p. 78).
*Morality* (11): Performer's ethical principles.
  “Bernard Robert is a Beethoven interpreter of sterling integrity” (Osborne, November 1995, p. 146).

### Performance evaluation criteria in critical review

**Figure 2:**
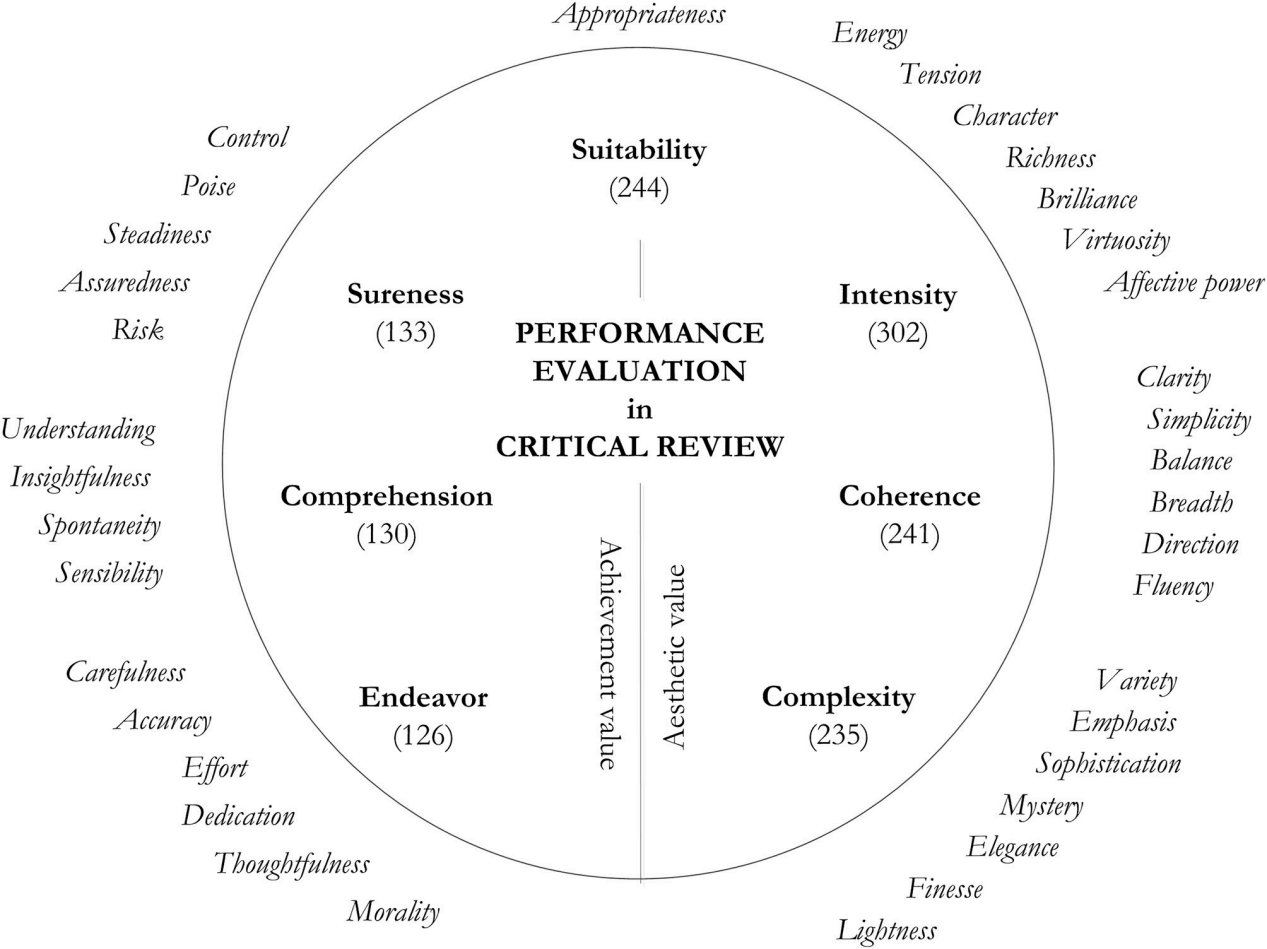
**Basic criteria of performance evaluation emerging from the analysis of descriptor-related value adding qualities in reviews**. Value adding qualities are shown outside the circle. Higher-order evaluation criteria are shown within the circle. Numbers in parentheses under criterion names indicate the frequency of occurrence of each criterion in the text.

On completion of the co-occurrence analyses between descriptors and valence loaded statements, 39 value adding qualities were identified that were used by critics to support their value judgments. Three of them (*Fast tempo, Slow tempo, Romantic* style) could be ascribed to their influence to other qualities (e.g., *Fast tempo* was praised for adding to *Energy* and *Fluency*, and criticized for marring *Clarity* and *Affective power*). Following Beardsley (1968), the remaining 36 value adding qualities were further analyzed and grouped into seven higher-order properties that were recurrently employed as criteria of value in critical review (Figure 2).

Figure 2 is subdivided in the center to indicate a distinction between the critical criteria. Three of the criteria on the right of the figure, **Intensity**, **Complexity**, and **Coherence**, are aesthetic related: they describe the (perceived) musical sound and how this is organized in time. Three more criteria on the left of the figure, **Sureness**, **Comprehension**, and **Endeavor**, are achievement related: they point at elements of the preparation and delivery of the performance that can be derived/assumed through an interpretation of what is heard, but that are not a description of the performance itself. One more criterion, **Suitability**, indicates the extent to which each of these criteria is desirable in a given musical context.

**Figure 3:**
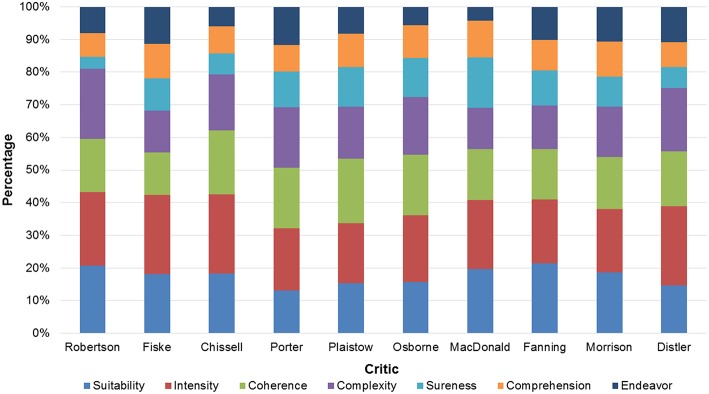
**Distribution of the seven evaluation criteria among critics**. For each critic, the relative frequency is shown with which each criterion was coded in the text.

Each review in our corpus discussed different recordings and performances. Discrepancies in the relative use of criteria could thus be linked to differences in the critic's individual evaluation and writing style as well as to the different nature of the recording and performance reviewed. Despite these confounding factors, the use of these seven criteria was consistently spread among critics (Kendall's coefficient of concordance *W* = 0.81, *p* < 0.001). Figure 3 shows the relative frequency with which each criterion was coded in the text, for each critic.

**Figure 4:**
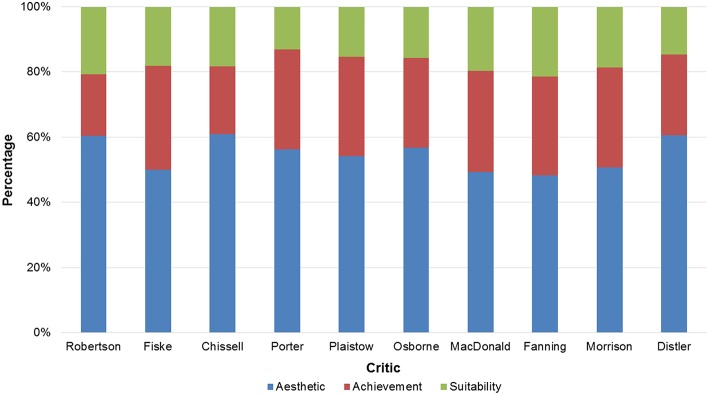
**Distribution of the seven evaluation criteria among critics, with aesthetic and achievement related criteria grouped together**. For each critic, the relative frequency is shown with which each criterion or group of criteria was coded in the text.

In particular, the proportion of Aesthetic, Achievement, and Suitability criteria was similar between critics: on average (median) each critic used in his/her reviews 55.15% (IQR = 50.17–59.45%) of Aesthetic, 30.39% (IQR = 25.56–30.78%) of Achievement, and 18.26% (IQR = 15.37–19.46%) of Suitability judgments (Figure 4).

In the present study, the analysis focused on the relationship between each performance descriptor and valence. However, tension emerged as a characterizing element of the relationship between criteria: Critics frequently discussed different value adding qualities as if they were interdependent, so that each quality only maintains its positive value as long as it is counterbalanced by—or it does not mar—other qualities. For example, an increase in **Complexity** in terms of *Emphasis* of musical details is appreciated, but only insofar as it does not taint *Fluency* and *Direction* of the performance:

“…only a few movements achieve the continuity and natural expressiveness proper to them …as if anxiety that no point should be missed has prompted him to underline and over-emphasize everything” (Plaistow, July 1966, p. 47).

A high level of **Intensity** is positive, but only as long as it does not mar *Clarity* or *Variety*:

“…in the final reckoning clarity of texture and articulation do get sacrificed on the altar of excitement, and this is rather a pity” (Plaistow, March 1964, p. 63).

“…such an attack on the first of the two movements seems, and is, effective enough at the time; but it does create less of a contrast with the turbulent second movement than is ideal” (MacDonald, March 1965, p. 57).

This interdependency between criteria is emphasized in Figure 2 by the circular shape. The term *Tightrope* was used to conceptualize this tension: performers in critical review are required to balance the criteria of value within the circle of evaluation. A successful equilibrium between these criteria was discussed in a universally positive light by the critics (*Tightrope* statements):

“…he balances sense and sensibility to an ideal degree” (Morrison, March 2003, p. 63).

“The great virtue of this player seems to me his combination of emotion and intellectuality” (Fiske, August 1963, p. 31).

“This is, indeed, the real Solomon: virtuosity married to the finest sensibility” (Osborne, November 2002, p. 86).

*Tightrope* statements were fairly common in critics' writing: a *post-hoc* analysis identified 42 instances of such statements spread across 31 out of the 100 reviews.

## Discussion

In the present study, we analyzed a large corpus of critical reviews of recorded performances to identify the reasons adduced to support value judgments. The findings have led to an empirically developed model of performance evaluation criteria in critical review that bears both pedagogical and theoretical implications.

In line with previous results (Alessandri et al., 2015), the majority of critics' statements (87.57%) were valence loaded, although the valence was typically mixed within each review, with a combination of positive, negative, and mixed statements. Alongside valence loaded judgments given in the canonical form “Performance P is good/bad because of feature F” numerous judgments were found in the form “P is X,” where X is a performance descriptor that also implies an evaluation by being inherently valence loaded. Despite the richness of performance aspects discussed in reviews—as reported by Alessandri et al. (2015)—the systematic analysis of valence loaded statements and their relationship with performance descriptors found that critics' evaluations resulted in a model comprising just seven evaluation criteria that were reliably used by all the critics in our review corpus.

Three of the newly identified critical criteria—**Intensity**, **Coherence**, and **Complexity**—relate to the aesthetic value of a performance. These criteria resonate with Kaplan and Kaplan's (1989) model of aesthetic appreciation of natural landscapes and with Beardsley's proposed triadic theory of aesthetic value in the arts (Beardsley, 1962, 1968, 1982). The latter proposes intensity, complexity, and unity as the only universally valid criteria of aesthetic value, such that an increase in one of them, all the rest being equal, will always add to the value of any given artwork. Although the present findings do not claim generalizability, they bear evidence as to the presence of these criteria in a large corpus of one genre of professional recorded performance criticism. As such, they support the centrality of these basic properties in our conceptualization and appreciation of music performance.

A comparison of the emergent model with McPherson and Schubert's (2004, p. 63–64) list of musical parameters commonly assessed in performance examinations in educational contexts shows that **Coherence** is a construct commonly scrutinized in education, in terms of the use of tempo, dynamics, and phrasing, as well as clarity in communicating structural and expressive musical features. **Intensity** and **Complexity**, by comparison, find only minor correspondence in McPherson and Schubert, suggesting a larger weight is given to these criteria in the assessment of professional performances, possibly linked to a stronger focus on craftsmanship over artistic value in educational assessments.

In addition to the aesthetic related properties, three more critical criteria emerged from the analysis, which assess the preparation and delivery of the performance as product of the performer's achievement: **Sureness**, **Comprehension**, and **Endeavor**. This finding clarifies the scope of the *Performer Qualities* themes identified by Alessandri et al. (2015). In this previous work, statements on assumed qualities of the performer such as their character, felt emotions, or intentions, emerged as a relevant constituent of critics' descriptions of performance. The present study builds on this report in finding that such statements are not just used to characterize the performance, but rather comprise a substantial portion of critics' value judgments. This conclusion supports Carroll's (2009) “success value” theory in aesthetics and emphasizes the importance of a “performer's achievement” for the appreciation of recorded piano performance.

The weight given to achievement-related properties in our model also raises questions on the extent to which the aesthetic value of a performance can be assessed in isolation. This goes to the heart of the debate in philosophy of art between empiricists and contextualists, the former claiming that artworks can be properly evaluated by relying solely on what can be perceived through the experience of the work; the latter affirming the necessity of integrating heard information with a series of thoughts and beliefs that go beyond the direct experience of the work (Beardsley, 1988; Currie, 1989; Davies, 2006; Graham, 2006). The present findings support a contextualist view, highlighting how considerations and assumptions about the performer play a strong role in the final assessment of a recording's value, tightly bound with aesthetic related criteria.

This in turn poses the question of how and to what extent these achievement-related criteria ought to be integrated into academic assessment protocols. In McPherson and Schubert (2004), **Sureness**, **Comprehension**, and **Endeavor** find partial correspondence in the parameters “confidence,” “accuracy,” “physical control,” and “understanding of style/overall structure/emotional character,” spread among the evaluation areas of communication, interpretation, expression and technique. Future studies may investigate the benefit of grouping these elements under a common achievement-related label for the reliability and perceived validity of educational assessments.

The six main critical criteria from our emergent model were used consistently by all ten critics across almost 90 years of reviews covering all of Beethoven's piano sonatas (100 recordings, at least six recordings for each of the 32 sonatas). However, the presence of the seventh criterion, **Suitability**, emphasized the context-dependency of value judgments in terms of musical, historical, and performance practice background. This finding supports Carroll's (2009) and Sibley's (see Dickie, 1987) context-aware generalism, advanced in response to critiques to Beardsley's theory. According to context-aware generalism, evaluation criteria are necessary in order to make a valid form of reasoned evaluation. These must be inherently positive or negative, and general enough to be valid within a given artistic context. In line with this, the present study identified value adding qualities that were used reliably in a large corpus of reviews within the context of recorded performance, for the chosen repertoire and cultural background.

Taken together, and in line with results from studies on inter-rater consistency in performance evaluation (Thompson et al., 1998; Kinney, 2009), the present findings suggest the possibility of relying—at least within the boundaries of a given repertoire and cultural matrix—on a few higher-order, inherently positive qualities that have more general applicability and intersubjective validity. The present account of evaluation criteria thus provides reference material that can be used in investigations of other review corpora, with the aim of clarifying discrepancies and commonalities in the evaluation of different repertoires, genres, and cultural contexts.

Finally, it is important to note that the tension found between criteria (*Tightrope*) suggested interdependency between performance properties such that a modification in one property may affect the perception and evaluation of others. The present study analyzed critics' evaluations as they are expressed in reviews, and not how and to what extent critics implemented any of the emergent criteria. As such, this finding has to be taken cautiously. Nonetheless, it is interesting to notice its concordance with the widely discussed “uniformity-in-variety” theory (Berlyne, 1971; McDermott, 2012) and interactionist perspectives on aesthetic response, like the processing fluency framework proposed by Reber et al. (2004). Together, these results call for a reflection on the nature of assessment schemes commonly used in music schools and in research. These schemes usually work under the assumption that the different properties identified by main and sub-criteria are independent from each other, so that it is ideally possible to achieve the highest mark in each of them. In our review corpus, however, critics discussed the different properties as if they were interdependent and accounted overtly for this interdependency in their assessments, such that certain combinations of performance qualities could achieve praise over and above the value of the single properties. Together with the aforementioned theories, these insights suggest that future developments in assessment schemes should embrace a perspective that accounts for the tension within properties in the conceptualization of performance value. As such, a successful performance is not the one that satisfies each of the criteria in a scheme, but rather the one that balances the properties in an ideal equilibrium, offering listeners a pleasing and engaging combination of elements. This perspective shifts the attention onto the relative weight of criteria in evaluation schemes, a topic long discussed in performance evaluation research (Mills, 1991), suggesting that a dynamic interplay exists between constructs. Future studies will have to explore how this more complex conceptualization of performance evaluation criteria can be translated into valid and reliable assessment tools.

To summarize, we analyzed a large corpus of critical reviews of Beethoven piano sonata recordings to explore reasons used in professional music criticism to support value judgments of performance. Analysis of the relationship between value judgments and performance descriptions led to the development of two sets of value adding qualities that were discussed by critics in the evaluation of primary (Musical parameters, Energy, Technique) and supervenient (higher order performance features like Style, Emotion, Character) performance descriptors. These lists of value adding qualities provide musicians with evidence of what expert critics focus on in their performance assessments and offer a practical resource for the preparation and evaluation of Beethoven sonatas, an essential part of each pianist's standard repertoire. These qualities have been summarized in a novel model of performance evaluation that identifies seven inherently positive higher-order properties of performance praised by critics. Tension between criteria also emerged as an important element of critics' judgments. This emergent model, drawn from *Gramophone* reviews of Beethoven's piano sonatas, provides empirical support for current theories of art criticism and aesthetic appreciation.

A great musical performance—according to our model—is one charged with power and technical precision as well as expressive intensity, rich in its complexity but unified and coherent. It is instilled with rigorous dedication and care; a performance that conveys a feeling of mastery, assurance, conviction, and a deep understanding of the music and the instrument. Above all, a successful performance is one that achieves balance between these elements while accounting for musical, historical and cultural frames.

## Author contributions

EA designed the study, collected, analyzed, and interpreted the data, and drafted the manuscript. VW contributed substantially to the study design and data analysis, critically revised the manuscript, and supervised the submission process. HE and AW contributed to the conception of the study and contextualization of findings and critically revised the manuscript. All authors approved the final version of the manuscript to be submitted for publication.

### Conflict of interest statement

The authors declare that the research was conducted in the absence of any commercial or financial relationships that could be construed as a potential conflict of interest. The reviewer, MO and handling Editor declared their shared affiliation, and the handling Editor states that the process nevertheless met the standards of a fair and objective review.

## Footnotes

^1^Table 3 lists 31 main and sub-descriptors. For **Musical Parameters** however, no statements were found that did not belong to any sub-descriptor, therefore there were in total 30 quote lists used in the analyses.

^2^Original review texts can be accessed through the *Gramophone* online archive, available as paid application for iPad, desktop or tablet. See www.gramophone.co.uk.
